# Cyst Fraction as a Biomarker in Autosomal Dominant Polycystic Kidney Disease

**DOI:** 10.3390/jcm12010326

**Published:** 2022-12-31

**Authors:** Larina A. Karner, Sita Arjune, Polina Todorova, David Maintz, Franziska Grundmann, Thorsten Persigehl, Roman-Ulrich Müller

**Affiliations:** 1 Department II of Internal Medicine and Center for Molecular Medicine Cologne, Faculty of Medicine and University Hospital Cologne, University of Cologne, 50937 Cologne, Germany; 2 Institute of Diagnostic and Interventional Radiology, University of Cologne, 50937 Cologne, Germany; 3 Cluster of Excellence: Cellular Stress Responses in Aging-Associated Diseases (CECAD), Faculty of Medicine and University Hospital Cologne, University of Cologne, 50937 Cologne, Germany

**Keywords:** ADPKD, MRI, cyst fraction

## Abstract

Autosomal dominant polycystic kidney disease (ADPKD) is the most common monogenic kidney disease. Patients at high risk of severe disease progression should be identified early in order to intervene with supportive and therapeutic measures. However, the glomerular filtration rate (GFR) may remain within normal limits for decades until decline begins, making it a late indicator of rapid progression. Kidney volumetry is frequently used in clinical practice to allow for an assessment of disease severity. Due to limited prognostic accuracy, additional imaging markers are of high interest to improve outcome prediction in ADPKD, but data from clinical cohorts are still limited. In this study, we examined cyst fraction as one of these parameters in a cohort of 142 ADPKD patients. A subset of 61 patients received MRIs in two consecutive years to assess longitudinal changes. All MRIs were analyzed by segmentation and volumetry of the kidneys followed by determination of cyst fraction. As expected, both total kidney volume (TKV) and cyst fraction correlated with estimated GFR (eGFR), but cyst fraction showed a higher R^2^ in a univariate linear regression. Besides, only cyst fraction remained statistically significant in a multiple linear regression including both htTKV and cyst fraction to predict eGFR. Consequently, this study underlines the potential of cyst fraction in ADPKD and encourages prospective clinical trials examining its predictive value in combination with other biomarkers to predict future eGFR decline.

## 1. Introduction

Autosomal dominant polycystic kidney disease (ADPKD) is the most common genetic cause of kidney failure and is characterized by the progressive enlargement of multiple bilateral renal cysts and a decline in kidney function [1,2,3,4]. The loss of kidney parenchyma due to cyst growth is continuous and irreversible in ADPKD [5]. However, the glomerular filtration rate (GFR) often remains within a normal range for many years, until a more rapid decline in kidney function usually occurs from the fourth decade of life onwards [6]. Curative therapeutic options for ADPKD do not yet exist. Current treatment approaches are mainly aimed at inhibiting renal cystogenesis and the resulting destruction of the kidney parenchyma [7], but can only modestly extend the time until kidney failure. Importantly, the beneficial effect depends on the time on therapy, making early identification of patients at high risk for severe disease progression an important asset. Precise outcome prediction enables optimized patient counseling and avoids the exposure of patients with slow disease progression to potential side effects. To date, various prognostic factors of ADPKD have entered clinical practice including genetic, clinical, and imaging information [8]. Total kidney volume (TKV)—usually assessed by MRI-based volumetry—is the most frequently used biomarker in ADPKD and, as such, can be considered the gold standard for the prediction of the outcome [9]. However, the mere use of volume disregards the fact that single large cysts can dominate this parameter without much of an impact on kidney integrity. Consequently, knowledge of the extent to which normal parenchyma has been replaced by cysts appears to be an important addition to the volume itself. Cyst fraction is not part of the clinical routine, largely due to missing data on its potential and the time-consuming nature of its assessment. Therefore, more evidence is urgently required to unveil the potential of this parameter. The study at hand examined cyst fraction in comparison to TKV, correlating both parameters to kidney function in a cohort of 142 ADPKD patients.

## 2. Materials and Methods

### 2.1. Study Population and Study Design

The study population was selected as a sample of the ADPKD Tolvaptan Treatment Registry (AD(H)PKD), a prospective multicenter observational study that documents clinical and laboratory parameters, medication intake, and current imaging data of study participants at annual intervals. The study is registered at clinicaltrials.gov (NCT02497521). AD(H)PKD enrolls adult patients (≥18 years) with ADPKD who present for evaluation of tolvaptan therapy. The study population described here included 142 participants in the AD(H)PKD Registry from October 2015 to December 2017. Further inclusion criteria were existing magnetic resonance imaging (MRI) of sufficient quality and availability of serum creatinine at the time of MRI, with a maximum time difference of four months. eGFR was calculated using the CKD-EPI equation (2009, Chronic Kidney Disease Epidemiology Collaboration) [10].

### 2.2. Magnetic Resonance Imaging

Kidney volume was measured by semi-automatic volumetric segmentation with subsequent manual editing in the software IntelliSpace Discovery (ISD, version 2.0, Philips Healthcare, Best, The Netherlands). The majority of MRI images were obtained using a T2-weighted Spectral Presaturation with an Inversion Recovery (SPIR) sequence. If not available, other sequences such as Fluid Attenuated Inversion Recovery (FLAIR) were used. After completion of the measurement of TKV, cyst volume was determined using the “Threshold Segmentation” plugin of ISD. For this, the AV-Score of the brightest area of the kidney parenchyma was measured, which then functioned as the lower masking threshold. The upper threshold was set at a signal intensity of 100,000. Cyst fraction was then calculated by dividing the resulting total cyst volume (TCV) by the TKV. The height-adjusted TKV (htTKV) was calculated for comparison to cyst fraction as the TKV divided by the patient’s height. Mayo classification was used to categorize ADPKD patients as previously described [11].

### 2.3. Statistics

Data management and data analysis were performed using IBM SPSS Statistics (version 26 and version 28, IBM Corp, Armonk, NY, USA). The normal distribution of the collected data was tested with the Kolmogorov–Smirnov test, histograms, and Q-Q plots. For normally distributed variables of an identical population at two different time points, the means of these were examined with a paired samples *t*-test. For non-normally distributed values, a Wilcoxon test was performed. A transformation with a logarithmic function was used when appropriate. As a non-parametric equivalent of analysis of variance to examine the central tendencies of independent samples, the Kruskal/Wallis test was performed. Here, the group-related differences between the medians of a variable were elicited to evaluate the distribution of the sample, and the effect size of intra-variable groups that showed a significant *p*-value was calculated for a more accurate assessment. The interaction of two variables concerning their monotonic relationship was determined using Spearman’s correlation coefficient and tested for significance. Univariate and multiple linear regression analyses were set up to examine the directional relationship between a dependent and several independent variables and to identify predictors. The residuals of the factors were all controlled for normal distribution and homoscedasticity. Multicollinearity was determined using multicollinearity diagnostics and the according variance inflation factors and tolerance levels, as well as a condition index <30 and the Pearson correlation coefficient. A *p*-value below 0.05 was considered statistically significant.

## 3. Results

### Baseline Characteristics

**Figure 1:**
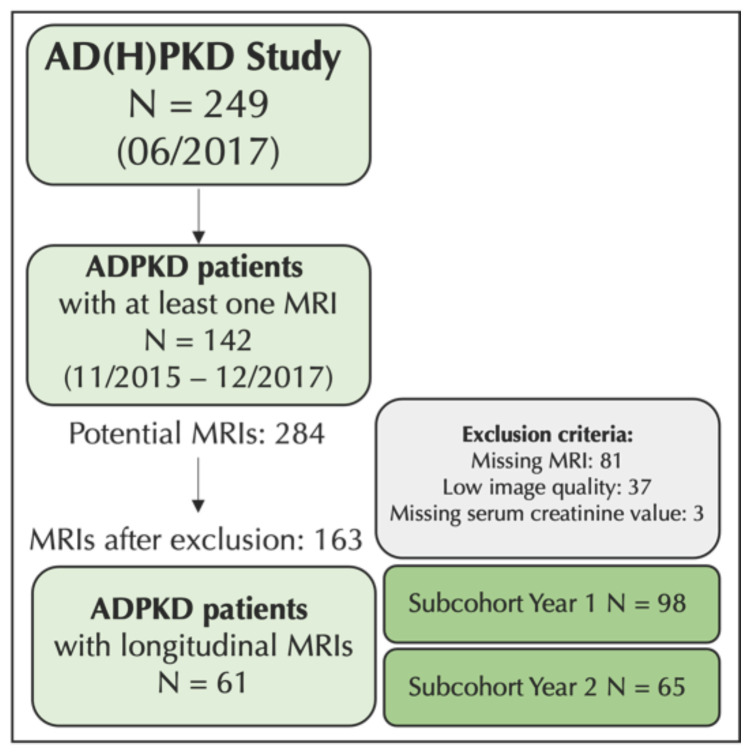
Study flow diagram depicting patient flow, exclusion criteria, and subgroup analyses. ADPKD, autosomal dominant polycystic kidney disease; MRI, magnetic resonance imaging.

At the initiation of the analyses at hand in 2017, the AD(H)PKD registry contained 249 ADPKD patients, 142 (63 men and 79 women) of which had undergone at least 1 MRI. This resulted in 284 visits, 163 of which were selected after the exclusion of missing MRI data (*n* = 81), low image quality (*n* = 37), or missing clinical values (*n* = 3) (Figure 1). A total of 61 (39% male) ADPKD patients had undergone longitudinal MRI exams, performed with an average time difference of 12 months (longitudinal cohort). Furthermore, patients’ characteristics are provided regarding 2 partly overlapping subcohorts, i.e., patients with an MRI in year 1 (*n* = 98, 41% male) on the one hand and in year 2 (*n* = 65, 40% male) on the other hand.

The mean age of the longitudinal ADPKD cohort of year 1 was 43 ± 12 years (range 23–67). The baseline characteristics of both cohorts are summarized in Table 1.

**Figure 2:**
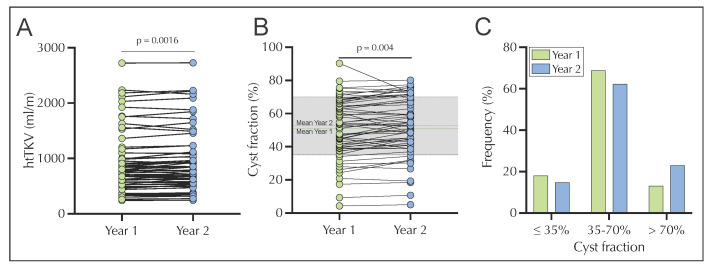
Longitudinal change in log htTKV (**A**) (year 1: mean 2.886 mL/m (green dotted line), year 2: 2.898 mL/m (blue dashed line)), cyst fraction (**B**) (year 1: mean 50.82% (green dotted line), year 2: 52.71% (blue dashed line)), and classification of the ADPKD cohort (*n* = 61) based on cyst fraction into low (≤35%), medium (35–70%), and high (>70%) in year 1 and in year 2 (**C**). The grey area in (**B**) depicts the medium cyst fraction (35–70%). htTKV, height-adjusted total kidney volume.

Analysis of the mean log htTKV revealed a significant increase (*p* < 0.001) over 1 year in the longitudinal cohort (Figure 2A). Similarly, the mean cyst fraction, which was defined as the cyst volume divided by the kidney volume, increased significantly (year 1: 50.82%; year 2: 52.71%; *p* = 0.004, Figure 2B). The cyst fraction was further divided into 3 groups, as previously described [12], and included participants with low cyst fraction values (≤ 35%), intermediate range (35–70%), and high values (>70%) (Figure 2C). In the longitudinal cohort and both subcohorts, the majority of study participants were classified as having an intermediate cyst fraction in both years. While the proportion of low and medium cyst fraction decreased between year 1 and 2, the proportion of high cyst fraction increased (Figure 2C and Supplemental Figure S1).

**Figure 3:**
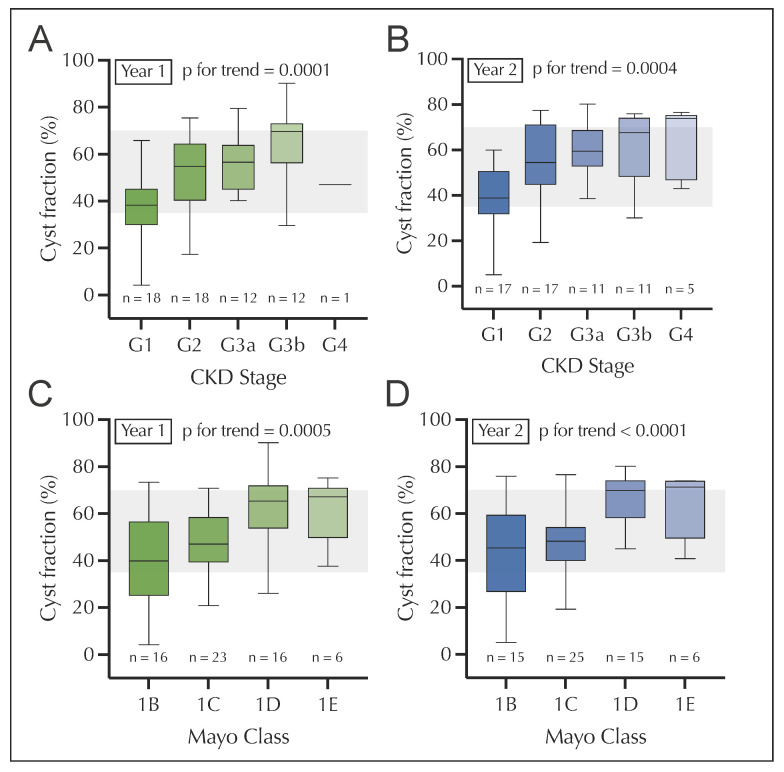
Cyst fraction (%) according to CKD stage (**A**,**B**) and Mayo class (**C**,**D**) among ADPKD patients for year 1 (**A**,**C**) and year 2 (**B**,**D**). CKD, chronic kidney disease. The grey area in (**A**–**D**) depicts the medium cyst fraction (35–70%).

When analyzing the distribution of the median cyst fraction according to CKD stages, we found a significant increase with increasing CKD stages in both years of the longitudinal cohort (Figure 3A). The same held true when analyzing Mayo classes 1B through E (Figure 3B).

**Figure 4:**
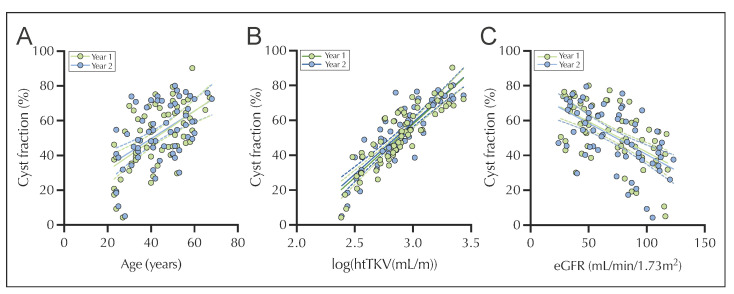
Correlation of cyst fraction with patients age ((**A**) year 1: ρ = 0.531, *p* < 0.001; year 2: ρ = 0.502, *p* < 0.001) log htTKV ((**B**) year 1: ρ = 0.881, *p* < 0.001 year 2: ρ = 0.832, *p* ≤ 0.001) and eGFR ((**C**) year 1: ρ = −0.573, *p* < 0.001, year 2: ρ = −0.560, *p* < 0.001) of the longitudinal cohort. Spearman correlation was performed for non-parametric data. eGFR, estimated glomerular filtration rate; htTKV, height adjusted total kidney volume.

In addition, a correlation analysis was performed for cyst fraction in the longitudinal cohort concerning age, log htTKV, and eGFR (Figure 4). Cyst fraction significantly correlated with both increasing age in years of the longitudinal cohorts (year 1 and 2, Figure 4A, year 1: ρ = 0.531, *p* < 0.001; year 2: ρ = 0.502, *p* < 0.001) and with log htTKV (Figure 4B, year 1: ρ = 0.881 year 2: ρ = 0.832 both with *p* < 0.001). Furthermore, cyst fraction was negatively correlated with kidney function (Figure 4C, year 1: ρ = −0.573, *p* < 0.001, year 2: ρ = −0.560, *p* < 0.001). The same associations were observed for the respective subcohorts (Supplemental Figures S2 and S3).

Despite the strong correlation between cyst fraction and htTKV, the scatterplot comparing these two parameters revealed two interesting “outlier” groups of patients. These groups showed fairly similar htTKV values while encompassing a large range of cyst fractions and vice versa. Since, within these groups, the two parameters may differ regarding their meaning for clinical severity (Supplemental Figure S4), their clinical characteristics were analyzed in more detail (Supplemental Tables S1 and S2). Subgroup 1 contained patients with a low cyst fraction (≤ 35%) and a mean htTKV of 310 mL/m while subgroup 2 contained patients with an average cyst fraction of 67–73% and an htTKV between 988 and 2235 mL/m (mean 1453 mL/m). Consequently, subgroup 1 consisted of young patients (mean age 32) with preserved kidney function and equal gender distribution and the vast majority of patients belonging to Mayo class 1B. The participants with the largest htTKV in this subgroup had a cyst fraction of 19% and an eGFR of 105 mL/min/1.73 m^2^. In subgroup 2, the average age of the patients was 49. The htTKVs of the two patients in this subgroup with the lowest eGFR (34 mL/min/1.73 m^2^) were vastly different. The difference between the two values was 1133 mL/m. In contrast, the cyst fraction was 71% and 69%, respectively.

After correlation analyses had established a relationship between cyst fraction and htTKV or kidney function (Figure 4), the influence of each value on eGFR was investigated using linear regression models (Table 2). For this analysis, we chose the subcohort at year 1, as this cohort contained the largest sample size (*n* = 98).

Both log htTKV and cyst fraction showed a highly significant relationship to eGFR in a univariate linear regression (Table 2). The model containing cyst fraction resulted in an adjusted R^2^ value of 0.366 (Model II), which was slightly higher than the R^2^ for the model containing log htTKV (Model I: adjusted R^2^ = 0.290). In an expanded regression model (Supplemental Table S3) with age and sex added as independent variables, it was shown that both log htTKV and cyst fraction remained significant.

To further elucidate the individual contribution of either cyst fraction or log htTKV on kidney function in ADPKD, we performed a multiple linear regression using a model that included both parameters with eGFR as the dependent variable (Model V, Table 3). Similar to model I, the analysis was conducted with subcohort year 1 (*n* = 98).

This model yielded almost the same adjusted R^2^ value of 0.362 as model II (Table 2). Only the cyst fraction remained significant, with a respective *p*-value of <0.001.

**Figure 5:**
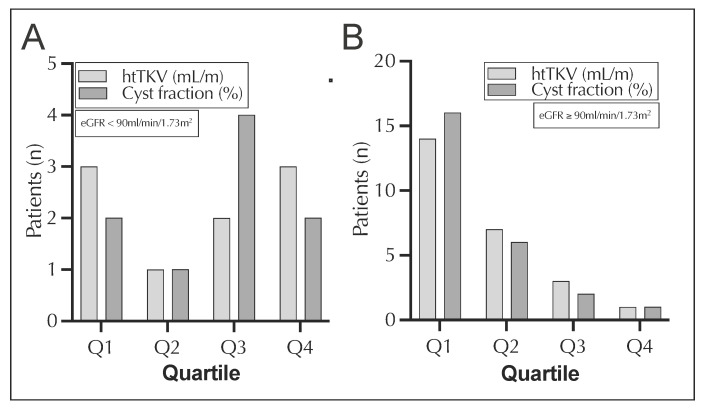
Quartiles of cyst fraction and htTKV of patients <40 years of the subcohort year 1 (*n* = 98) for either eGFR <90 (**A**) or ≥90 (**B**) mL/min/1.73 m^2^. eGFR, estimated glomerular filtration rate; htTKV, height adjusted total kidney volume.

Furthermore, considering the special importance of predictive biomarkers in young patients, an analysis of participants below 40 years of age (*n* = 34) was performed in subcohort year 1. These patients were divided into two categories according to whether they had a normal (≥90 mL/min/1.73 m^2^) or reduced (<90 mL/min/1.73 m^2^) eGFR. The group with the normal eGFR contained 9 patients, and the group with the reduced kidney function had 25 patients. We hypothesized that patients with intact kidney function would be more likely to be in the first or second quartile. Conversely, a higher number of individuals with lower eGFR would be in the third or fourth quartile. Of the patients with an eGFR < 90 mL/min/1.73 m^2^, five participants were in the top quartiles of htTKV and six were in those of the cyst fraction, while there was one more patient in the first quartile of the htTKV than in the cyst fraction (Figure 5A).

Figure 5B shows that the cyst fraction in the group of individuals with an eGFR of ≥90 mL/min/1.73 m^2^ placed two more patients in the first quartile than the htTKV. In contrast, there were only three individuals in the third and fourth quartiles of the cyst fraction, whereas there was a total of four in those of the htTKV.

## 4. Discussion

Prognostic imaging parameters of ADPKD have become the gold standard for patient selection concerning therapeutic measures [7,9]. Disease severity is associated with an increase in kidney volume [13]. Adjustments of TKV to height showed that even a single examination of the htTKV can predict the risk of disease progression [14]. However, because of the possibility of kidney atrophy/fibrosis, kidney volume alone is not always informative [11]. Besides, single dominating cysts have a high impact on TKV while hardly affecting the parenchyma.

It is important to note that kidney volume has to be regarded in the context of age and sex. The Mayo classification—the TKV-based clinical standard tool for outcome prediction—divides patients into classes 1A–E and 2, respectively, and is based on patients’ TKV, height, and age. It is used to predict the future decline of eGFR [11]. However, despite its high value in clinical routine, it may underestimate or overestimate the progression tendency of patients due to the drawbacks of kidney volume as its underlying parameter. Some patients in classes 1A/1B have a higher-than-expected eGFR loss per year, despite their classification in the low-risk category, whereas an unexpectedly low eGFR loss may be observed in some patients in classes 1C/1D/1E. This is partly because the TKV does not reflect proportionate cyst involvement in the total volume, but rather measures the volume of the kidney as a whole, making it impossible to conclude cyst distribution or number, and thus intact parenchyma. Consequently, any merely TKV-based approach would come with an inherent limit to the precision. Cyst fraction, which represents the proportion of cystically altered kidney tissue relative to healthy parenchyma, may add more information in kidney imaging.

In our study, cyst fraction increased significantly by 2% within approximately 1 year (+/− 4 months) in the longitudinal cohort. This is consistent with the results of two other CT-based studies with smaller cohorts, in which an increase in cyst fraction of 1.96% and 2% was found, respectively [15,16]. In 2011, a study of normotensive and hypertensive children with ADPKD found a significant increase in body surface-adjusted cyst fraction of approximately 4.7%/year in the hypertensive group and 1.7%/year in the non-hypertensive group [17]. Regarding the association between cyst fraction and log htTKV, a strong positive correlation (ρ = 0.832 to 0.881) was found, a finding that is in line with both pathophysiological assumptions and previous data [12,18]. It has to be noted that both cyst fraction and htTKV are calculated based on TKV, obviously resulting in a certain degree of collinearity (Supplemental Table S4). The relationship between kidney function and cyst fraction (ρ = −0.573 to −0.560) or age (ρ = 0.531 to 0.502) was moderately strong for both variables, with a slightly stronger correlation with eGFR for cyst fraction than for htTKV in the longitudinal cohort at year 1. The moderately strong correlation between eGFR and cyst fraction or htTKV has also been demonstrated in other studies [18,19].

When the two variables were further examined together in univariate and multiple linear regression analysis, cyst fraction was always found to be an independent predictor of kidney function (B = −0.854; β = −0.538; *p* < 0.001). The beta coefficients showed a higher value for the cyst fraction, indicating their superiority over htTKV. However, the limited R^2^ of both the htTKV and—even though slightly higher—the cyst fraction containing model point towards the fact that a combination of biomarkers will always be required to predict future outcomes in ADPKD (e.g., genotype, serum markers such as copeptin, etc.).

A recent study by Riyahi et al. also identified renal cyst fraction to be associated with disease severity and, importantly, as an independent predictor of future eGFR [18]. In contrast to their study, we found log htTKV as an independent predictor only in the univariate but not in the multiple regression analyses. Unfortunately, a comparison of the regression coefficients B and beta coefficients of said study by Riyahi et al. to determine which of the two variables had a stronger effect on eGFR was not available. Interestingly, they identified additional imaging parameters improving the model, including hemorrhagic cysts as the most potent predictor. To prove that a one-time examination of both the cyst fraction and hemorrhagic cysts would predict future GFR and truly add to existing predictive models, a prospective study in more patients would be an important next step now. Considering the variability of eGFR, it is important that such a study collects a sufficient number of measurements over several years.

Another known prognostic factor of ADPKD is gender, with males being predisposed to severe disease progression [20]. A relationship between gender and cyst expansion rate of ADPKD patients was previously described [21]. In contrast, in a recently published study, no significant relationship was found between size-adjusted total cyst volume or total cyst count and gender in ADPKD [22]. In a study by Yin et al., larger kidney cyst fractions were associated with a higher likelihood of inferior vena cava compression by cysts, with men showing a higher prevalence than women in this regard, even though both genders had a similar cyst fraction [23]. In our study, we did not detect an association between cyst fraction and the gender of the study participants (Supplemental Table S5). In contrast, however, a significant relationship prevailed between htTKV and gender (η = 0.266 to 0.284, Supplemental Table S5).

The subgroup analyses also point towards a potentially higher discriminatory power of cyst fraction compared with the htTKV concerning disease severity. In the subcohort of individual participants with a similar htTKV and a large range of cyst fraction, the individual with the lowest eGFR and the highest cyst fraction had a rather low htTKV. In contrast, the patient in this subgroup with the largest value of htTKV yielded normal kidney function. Within the subgroup of patients with a similar cyst fraction and a large range of htTKV, two individuals had a high cyst fraction and a low eGFR. When contrasted, they differed greatly in their htTKVs. However, the cyst fraction was found to be high in both patients, so it could be concluded that this was more indicative of eGFR than the htTKV. The subgroup analysis of patients <40 years of age and distribution to TKV/cyst fraction quartiles could strengthen this view. However, admittedly, whether cyst fraction has an added value for such subgroups of patients, the prognosis of whom would be misinterpreted based on TKV, cannot be finally answered due to low numbers and will require additional data.

### Limitations

Limitations concerning the study population are that a large proportion of patients could be assigned to CKD stage G1. Consequently, the potential of the cyst fraction for estimating the severity of the disease is likely significantly understated in our study.

In addition, data were collected and analyzed at only two distinct time points in this study. Although cyst fraction was identified as an independent predictor of simultaneous kidney function in the univariate and multivariate linear regression analyses, additional research is still required to make a statement about its predictor function on future eGFR. Besides, TKV and cyst fraction come with collinearity, a fact that has to be kept in mind when interpreting the data. In addition, our sample consisted of only 98, 65, and 61 patients, respectively. To make a definitive statement regarding the predictor function of the cyst fraction, it will be necessary to investigate a larger sample size in which future eGFR slopes—preferably over several years—can be predicted.

## 5. Conclusions

This study adds to the existing evidence on the potential of cyst-fraction, adding important data to TKV. These findings should now be the basis for designing prospective studies focusing on future eGFR prediction in larger cohorts and combination with other imaging and non-imaging biomarkers. Moreover, it will be crucial to continue the development of tools allowing for simple measurement of the cyst fraction to enable clinical use in the future.

## Figures and Tables

**Table 1:** Baseline characteristics of the ADPKD cohort. CKD, chronic kidney disease; eGFR, estimated glomerular filtration rate; htTKV, height adjusted total kidney volume; SD, standard deviation.

	Longitudinal Cohort		Subcohort I	Subcohort II
Year	Year 1	Year 2	Year 1	Year 2
n (male %)	61 (39%)		98 (41%)	65 (40%)
Age (years) mean ± SD	43 ± 12	44 ± 12	43 ± 12	44 ± 12
eGFR (mL/min/1.73 m^2^), mean ± SD	71 ± 28	69 ± 29	73 ± 28	69 ± 28
htTKV (mL/m), mean ± SD	914 ± 567	935 ± 569	901 ± 528	946 ± 553
Mayo Classification, *n*	61		98	65
1A	0	0	0	0
1B	16	15	29	15
1C	23	25	35	26
1D	16	15	23	18
1E	6	6	11	6
CKD stage, *n*	61		98	65
1 eGFR > 90 mL/min/1.73 m^2^	18	17	30	17
2 eGFR 60–89 mL/min/1.73 m^2^	18	17	31	18
3a eGFR 45–59 mL/min/1.73 m^2^	12	11	19	14
3b eGFR 30–44 mL/min/1.73 m^2^	12	11	16	11
4 eGFR 15–29 mL/min/1.73 m^2^	1	5	2	5
5 eGFR < 15 mL/min/1.73 m^2^	0	0	0	0

**Table 2:** Univariate linear regression analysis of subcohort year 1. eGFR served as the dependent variable, while the cyst fraction, or log htTKV, acted as the independent variable. eGFR, estimated glomerular filtration rate; DF, degrees of freedom; htTKV, height-adjusted total kidney volume.

**Model I: eGFR ~ log htTKV**						
**DF:** 1**; Adjusted R^2^:** 0.290						
	**Regression coefficient B**	**Std. Error**	**ß**	**|T|**	***p***	***p*-value summary**
Constant	249.305	27.718		8.994	<0.001	****
Log htTKV	−61.064	9.575	−0.546	−6.377	<0.001	****
**Model II: eGFR ~ cyst fraction**						
**DF:** 1**; Adjusted R^2^:** 0.366						
	**Regression coefficient B**	**Std. Error**	**ß**	**|T|**	***p***	***p*-value summary**
Constant	121.618	6.799		17.887	<0.001	****
Cyst fraction	−0.969	0.128	−0.611	−7.554	<0.001	****

****: *p* value of <0.001.

**Table 3:** Multivariate linear regression of the subcohort year 1 (*n* = 98). eGFR served as the dependent variable, while the cyst fraction and log htTKV acted as the independent variables. eGFR, estimated glomerular filtration rate; DF, degrees of freedom; htTKV, height-adjusted total kidney volume; ns, not significant.

Model V: eGFR ~ log htTKV + Cyst Fraction						
DF: 2; *p* < 0.001; Adjusted R^2^: 0.362						
	Regression Coefficient B	Std. Error	ß	|T|	*p*	*p*-Value Summary
**Constant**	143.299	40.612		3.528	<0.001	****
**Log htTKV**	−9.520	17.579	−0.085	−0.542	0.589	ns.
**Cyst fraction**	−0.854	0.249	−0.538	−3.424	<0.001	****

****: *p* value of <0.001.

## Data Availability

Not applicable.

## Supplementary Materials

The following supporting information can be downloaded at: https://www.mdpi.com/article/10.3390/jcm12010326/s1, Figure S1: Classification of the ADPKD subcohorts for year 1 (*n* = 98) and year 2 (*n* = 65) based on cyst fraction into low (≤ 35%), medium (35–70%), and high (>70%). Figure S2: Cyst fraction (%) according to CKD stage (A,B) and Mayo class (C,D) among ADPKD subcohorts for year 1 (*n* = 98) (A,C) and year 2 (*n* = 65) (B,D). The grey area in (A–D) depicts the medium cyst fraction (35–70%). Figure S3: Correlation of cyst fraction with eGFR (A,B) and log htTKV (C,D) for the ADPKD subcohort for year 1 (*n* = 98) and year 2 (*n* = 65). eGFR estimated glomerular filtration rate; htTKV, height-adjusted total kidney volume. Figure S4: Subgroup analysis of subcohort year 1 (*n* = 98). Red rectangles indicate subgroups. Subgroup 1 contained all patients with a cyst fraction below or equal to 35%, while subgroup 2 contained all patients with a cyst fraction between 67 and 73%. The grey area depicts the medium cyst fraction (35–70%). CF, cyst fraction; htTKV, height-adjusted total kidney volume. Table S1. Subgroup 1 of patients with similar htTKV and a large spread of the cyst fraction. eGFR, estimated glomerular filtra-tion rate; htTKV, height-adjusted total kidney volume. Table S2. Subgroup 2 of patients with similar cyst fraction and a large spread of the htTKV. eGFR, estimated glomerular filtra-tion rate; htTKV, height-adjusted total kidney volume. Table S3. Multivariate linear regression of the subcohort year 1 (*n* = 98). eGFR served as the dependent variable, while the age, sex, and log htTKV or cyst fraction acted as the independent variables. eGFR, estimated glomerular filtration rate; DF, degrees of freedom; htTKV, height-adjusted total kidney volume; ns, not significant. Table S4. Collinearity analysis for model V (multivariate linear regression, Table 3). eGFR served as the dependent variable. DF, degrees of freedom; htTKV, height-adjusted total kidney volume. Table S5. Eta correlation was performed between gender and cyst fraction (η = not significant) or htTKV (η = 0.266 to 0.284). htTKV, height-adjusted total kidney volume. * signifies a *p*-value of <0.05 = *, ** signifies a *p*-value of <0.01.
