# Supplementary material for: Cyst Fraction as a Biomarker in Autosomal Dominant Polycystic Kidney Disease
Source: J Clin Med. 2022 Dec 31;12(1):326. doi: 10.3390/jcm12010326 (PMC9821598; doi:10.3390/jcm12010326)
Supplement: Supplementary file 1 [file jcm-12-00326-s001.zip › jcm-2076022-supplementary.pdf]

Journal of
Clinical Medicine

MDPI

Supplementary Materials: The following supporting information can be downloaded at: https://www.mdpi.com/article/10.3390/jcm12010326/s1,

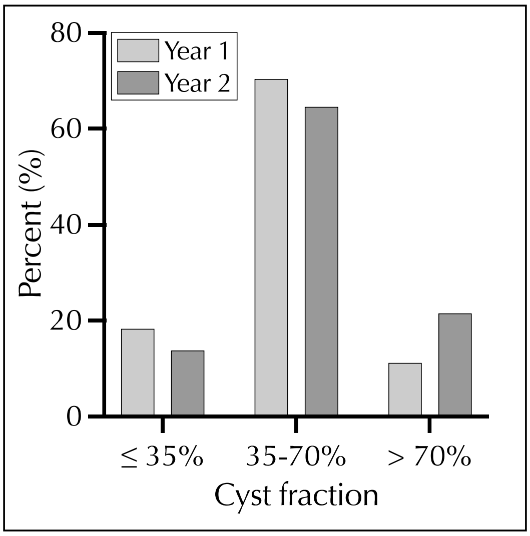

Figure S1. Classification of the ADPKD subcohorts for year 1 (n = 98) and year 2 (n = 65) based on cyst fraction into low (≤ 35%), medium (35-70%), and high (> 70%).

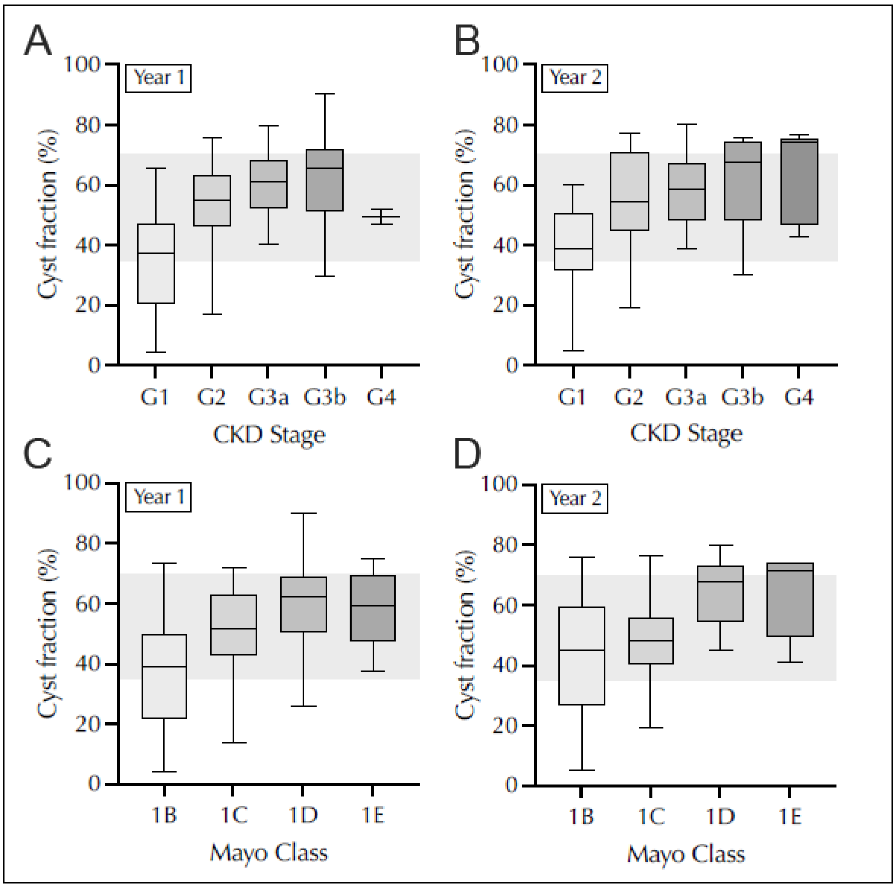

Figure S2. Cyst fraction (%) according to CKD stage (A,B) and Mayo class (C,D) among ADPKD subcohorts for year 1 (n = 98) (A,C) and year 2 (n = 65) (B,D). The grey area in (A)-(D) depicts the medium cyst fraction (35-70%).

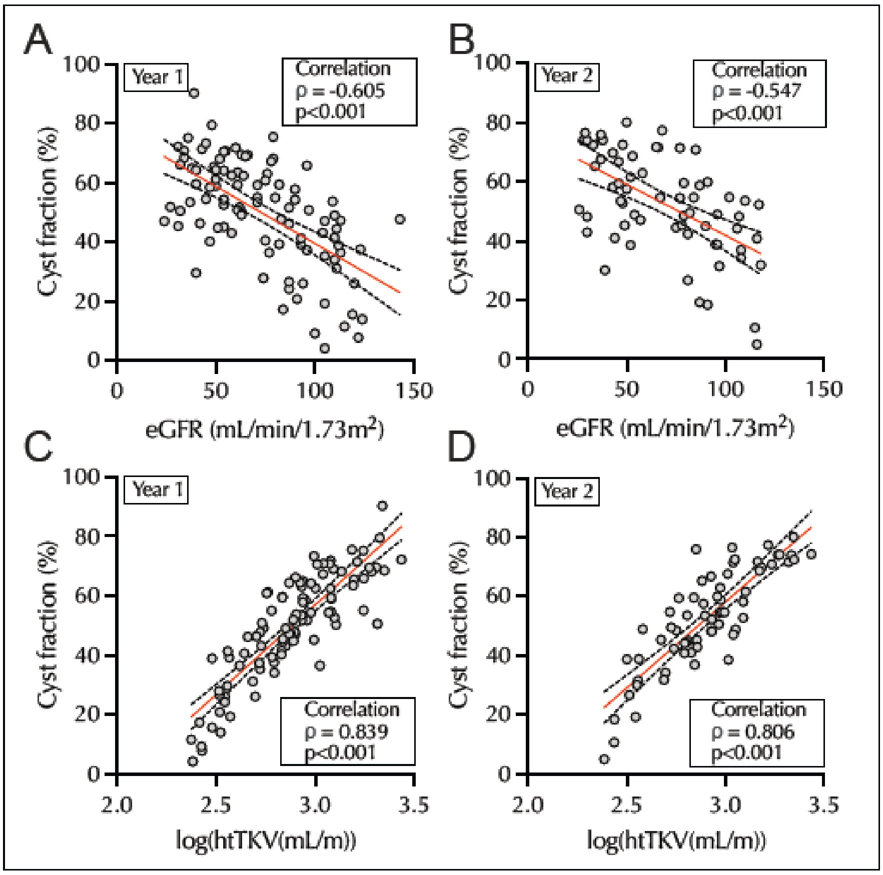

Figure S3. Correlation of cyst fraction with eGFR (A,B) and log htTKV (C,D) for the ADPKD subcohort for year 1 (n = 98) and year 2 (n = 65). eGFR, estimated glomerular filtration rate; htTKV, height-adjusted total kidney volume.

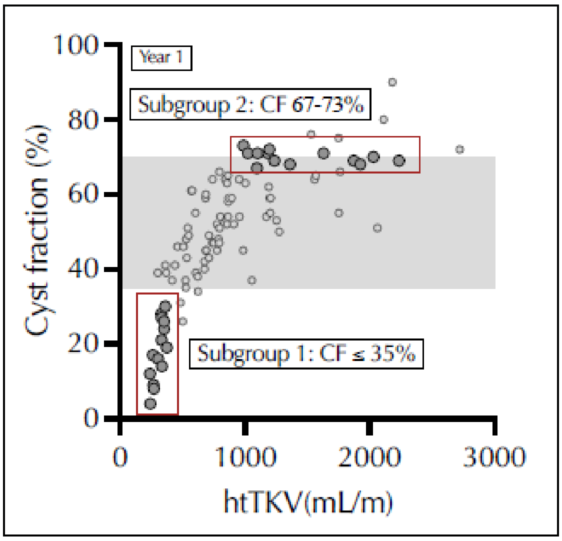

Figure S4. Subgroup analysis of subcohort year 1 (n = 98). Red rectangles indicate subgroups. Subgroup 1 contained all patients with a cyst fraction below or equal to 35%, while subgroup 2 contained all patients with a cyst fraction between 67-73%. The grey area in depicts the medium cyst fraction (35-70%). CF, cyst fraction; htTKV, height-adjusted total kidney volume.

Table S1. Subgroup 1 of patients with similar htTKV and a large spread of the cyst fraction. eGFR, estimated glomerular filtration rate; htTKV, height-adjusted total kidney volume.

Pat.-ID	Gender (male/female)	Age (years)	eGFR (mL/min/1,73m²)	htTKV (mL/m)	Cyst fraction (%)	Mayo Class (1A-1E)
**1**	f	52	40	360	30	1B
**2**	f	41	74	328	28	1B
**3**	f	23	84	261	17	1B
**4**	f	38	87	330	27	1B
**5**	m	40	87	350	24	1B
**6**	m	23	91	330	21	1C
**7**	m	46	94	350	26	1B
**8**	m	24	100	267	9	1B
**9**	f	27	105	242	4	1B
**10**	m	34	105	373	19	1B
**11**	f	18	115	238	12	1B
**12**	m	27	119	303	16	1B
**13**	f	26	122	269	8	1B
**14**	m	25	124	335	14	1C
**Mean**	n.a.	32	96	310	18	n.a.

Table S2. Subgroup 2 of patients with similar cyst fraction and a large spread of the htTKV. eGFR, estimated glomerular filtration rate; htTKV, height-adjusted total kidney volume.

Pat.-ID	Gender (male/female)	Age (years)	eGFR (mL/min/1,73m²)	htTKV (mL/m)	Cyst fraction (%)	Mayo Class (1A-1E)
**15**	f	55	34	1102	71	1C
**16**	m	30	34	2235	69	1E
**17**	m	50	43	1630	71	1D
**18**	f	67	45	988	73	1B
**19**	m	52	52	1926	68	1D
**20**	f	51	54	1024	71	1C
**21**	f	63	55	1184	71	1C
**22**	f	51	60	1196	72	1C
**23**	m	42	63	2031	70	1E
**24**	m	46	65	1237	69	1D
**25**	m	41	66	1874	69	1E
**26**	m	44	78	1096	67	1D
**27**	f	39	79	1360	68	1D
**Mean**	n.a.	49	56	1453	70	n.a.

Table S3. Multivariate linear regression of the subcohort year 1 (n = 98). eGFR served as the dependent variable, while the age, sex and log htTKV or cyst fraction acted as the independent variables. eGFR, estimated glomerular filtration rate; DF, degrees of freedom; htTKV, height-adjusted total kidney volume; ns, not significant.

Model III: eGFR ~ age + sex + log htTKV						
**DF:** 3; **p** < 0.001; **Adjusted R^2:** 0.570						
	**Regression coefficient B**	**Std. Error**	**ß**	**|T|**	**p**	**p-value summary**
**Constant**	229.114	21.714		10.551	< 0.001	****
**Age**	−1.381	0.179	−0.590	−7.723	< 0.001	****
**Sex**	−0.184	4.060	−0.003	−0.045	0.964	n.s.
**Log htTKV**	−33.425	8.636	−0.299	−3.870	< 0.001	****

Model IV: eGFR ~ age + sex + cyst fraction						
**DF:** 3; **p** < 0.001; **Adjusted R^2:** 0.561						
	**Regression coefficient B**	**Std. Error**	**ß**	**|T|**	**p**	**p-value summary**
**Constant**	157.191	9.678		16.242	< 0.001	****
**Age**	−1.303	0.196	−0.556	−6.658	< 0.001	****
**Sex**	−3.423	3.926	−0.060	−0.872	0.385	n.s.
**Cyst fraction**	−0.466	0.131	−0.293	−3.557	< 0.001	****

Table S4. Collinearity analysis for model V (multivariate linear regression, Table 3). eGFR served as the dependent variable. DF, degrees of freedom; htTKV, height-adjusted total kidney volume.

**Model**	**Dimension**	**Eigenvalue**	**Condition index**	**Variance proportions:**		
				**Constant**	**Log htTKV (mL/m)**	**Cyst fraction (%)**
**V**	1	2.935	1.000	0.00	0.00	0.00
	2	0.064	6.795	0.01	0.00	0.30
	3	0.001	49.451	0.99	1.00	0.69

Table S5. Eta correlation was performed between gender and cyst fraction (η = not significant) or htTKV (η = 0.266 to 0.284). htTKV, height-adjusted total kidney volume. * signifies a p-value of < 0,05 = *, ** signifies a p-value of < 0,01.

		**Gender (male/female)**
**Cyst fraction (%)**	Longitudinal cohort Year 1 (n = 61)	η = n.s.
	Longitudinal cohort Year 2 (n = 61)	η = n.s.
	Subcohort Year 1 (n = 98)	η = n.s.
	Subcohort Year 2 (n = 65)	η = n.s.
**htTKV (mL/m)**	Longitudinal cohort Year 1 (n = 61)	η = 0.284*
	Longitudinal cohort Year 2 (n = 61)	η = 0.275*
	Subcohort Year 1 (n = 98)	η = 0.266**
	Subcohort Year 2 (n = 65)	η = 0.270*
